# Cluster-randomized trial of a web-assisted tobacco quality improvement intervention of subsequent patient tobacco product use: a National Dental PBRN study

**DOI:** 10.1186/1472-6831-13-13

**Published:** 2013-02-23

**Authors:** Thomas K Houston, Kathryn L DeLaughter, Midge N Ray, Gregg H Gilbert, Jeroan J Allison, Catarina I Kiefe, Julie E Volkman

**Affiliations:** 1 VA Bedford Medical Center, Bedford, MA, USA; 2 University of Massachusetts Medical School, Worcester, MA, USA; 3 University of Alabama at Birmingham, Birmingham, AL, USA

**Keywords:** Tobacco use cessation, Dental practice, Health services research

## Abstract

**Background:**

Brief clinician delivered advice helps in tobacco cessation efforts. This study assessed the impact of our intervention on instances of advice given to dental patients during visits on tobacco use quit rates 6 months after the intervention.

**Methods:**

The intervention was cluster randomized trial at the dental practice level. Intervention dental practices were provided a longitudinal technology-assisted intervention, oralcancerprevention.org that included a series of interactive educational cases and motivational email cues to remind dental provides to complete guideline-concordant brief behavioral counseling at the point of care. In all dental practices, exit cards were given to the first 100 consecutive patients, in which tobacco users provided contact information for a six month follow-up telephone survey.

**Results:**

A total of 564 tobacco using dental patients completed a six month follow-up survey. Among intervention patients, 55% reported receiving advice to quit tobacco, and 39% of control practice patients reported receiving advice to quit tobacco (p < 0.01). Six-month tobacco use quit rates were not significantly between the Intervention (9%) and Control (13%) groups, (p = 0.088).

**Conclusion:**

Although we increased rates of cessation advice delivered in dental practices, this study shows no evidence that brief advice by dentist’s increases long-term abstinence in smokers.

**Trial registration:**

ClinicalTrials.gov NCT00627185

## Background

Despite widespread acceptance that tobacco use is the primary preventable cause of death; rates of this risky behavior have not substantially declined in the past decade [1]. The Centers for Disease Control and Prevention (CDC) estimates that approximately 46.6 million U.S. adults smoke cigarettes, and other use smokeless tobacco [1]. The 2006 State of the Science Conference Statement on Tobacco Use noted that several interventions to enhance tobacco cessation are under-utilized [2]. In particular, the use of brief provider advice is effective, but under-implemented especially in specialized healthcare settings. Advice from healthcare providers can help people who use tobacco products to quit and maintain quit rates [3]. Rates of implementing brief advice vary, depending on clinical setting. Primary care physicians reported advising 94.9% of smokers to stop smoking, whereas dental providers advising 70.6% of smokers to quit [4]. Thus, dental practices have room for improvement and have been targeted by quality improvement programs.

The dental visit represents a unique opportunity for tobacco control, for a variety of reasons. First and especially in today’s economic climate, some tobacco users do not regularly visit medical providers, but may seek dental care for routine cleanings and cosmetic reasons [5]. Specifically, adults aged 20–44 rarely visit their physicians for preventive care, while more than half of adult smokers visit a dentist each year [5]. As tobacco use causes cosmetic and oral health problems, smokers may be concentrated in this population seeking dental, but not medical care.

In addition, dentists have direct access to view and convey risks of tobacco use. The end organ damage from cigarettes (e.g. cardiovascular disease and pulmonary cancer) is mostly long-term, unseen, and silent until after damage has occurred. Because dentists work with teeth and gums, they directly observe the impact of tobacco use. Cosmetic damage such as staining and diseases such as periodontal diseases are most likely to be addressed in dental visits. These impacts can then in turn be conveyed to the tobacco user - at the point of care - providing the unique opportunity to “see” what smoking or smokeless tobacco is doing to their body. Dentists can make quit tobacco advice personally relevant by including information that is most important and pertinent to the patient such as yellowing of teeth [6]. In addition, mounting evidence supports the necessity of good oral health to maintain optimum general health [7].

Tobacco control quality improvement programs designed to increase tobacco product use cessation advice in dental practice has been successful [8]. Interventions that have targeted dentists or hygienists, have involved workflow support, academic detailing, patient education materials, and multimedia interventions. One such web-assisted tobacco control quality improvement program for dental practice was delivered using http://www.dtc.cme.uab.edu. This multi-statecommunity-based intervention targeted dentists and dental staff (hygienists and dental assistants) in 140 dental practices. Oralcancerprevention.org included case-based education to train dental providers on how to provide quit tobacco product advice at the point of care, individualized practice improvement planning, workflow support materials, and patient education quit materials. In previous publications from this NIH-funded study, we demonstrated that this web-assisted multi-component intervention resulted in an 11% increase in tobacco use cessation advice in dental practices, significantly greater improvement than control (p < 0.04) [9]. However, to date, no tobacco control quality improvement programs targeting dental providers have evaluated the impact of practice improvement on the subsequent behavior of tobacco users seen in the dental practices.

We used new, longitudinal six-month follow-up data on individual dental patients who used tobacco products and sought care in http://www.dtc.cme.uab.edu intervention and control practices participating in our national cluster-randomized trial. By improving the volume and quality of brief tobacco advice in dental practices, we hypothesized that dental tobacco users recipients of the improved care would be more likely to change their behavior. Thus, there would be higher cessation rates among those who received enhanced tobacco cessation counseling from their intervention dental provider, compared to those patients in the usual care control group at six month follow-up.

## Methods

### Study design

We conducted a cluster-randomized trial (2005–2008) to evaluate http://www.dtc.cme.uab.edu, a web-assisted tobacco control quality improvement program designed to increase the quality and quantity of tobacco product cessation advice delivered at the point of care by dental providers. A total of 143 participating practices were randomized to either a provider-facingweb-delivered multimodal educational intervention or usual-care wait list control, and completed follow-up data collection. Dental patients who used tobacco products were recruited from the http://www.dtc.cme.uab.edu quality improvement program intervention and control practices. These dental patients consented to participate in six-month follow-up calls to report their tobacco use behavior after receiving dental care. This evaluation was funded by the National Institutes of Health (National Institute of Drug Abuse and the National Institute on Dental and Craniofacial Research), and was approved by the University of Alabama at Birmingham Institutional Review Board (IRB).

### Clinical setting and provider sample

Community-based dental practices were recruited from Alabama, Georgia, Florida and North Carolina, identified using dental licensure lists and mailing lists from the National Dental PBRN, a dental practice-based research network [10]. These were community-based dental practices with varying numbers of providers and varying setting, rural and urban. Accounting for clustering of patients within practices, a sample size of 130 practices (65 per arm) was needed to detect a 10% increase in tobacco use cessation advice, comparing intervention and control. Practices were randomized to the intervention or control groups using a permuted block randomization sequence generated by our biostatistician. Of the randomized practices who provided follow-up data (N=143; 70 intervention practices, 73 control practices), 79.2% were solo dental practices, 94.5% were general practice dentistry (others included a periodontal practice) and 78.1% had 4 or less dental hygienist or assistants.

### The OralCancerPrevention.org intervention

In the tobacco control quality improvement program intervention practice providers were sent information and instructions to log on to OralCancerPrevention.org. This interactive web-assisted system included educational cases, patient education and practice tools, a forum for chatting, opportunities to ask questions, and a presentation of headlines, with pushed email educational reminders to cue participation [9], see Additional file 1 for screenshots of the website. Developed by a team of dentists, hygienists, tobacco and health informatics experts, the http://www.dtc.cme.uab.edu website provided the dental practices with strategies for advising patients on tobacco control [9]. Tobacco users in these practices thus received usual dental care and had the opportunity to receive the guideline-concordant tobacco cessation advice provided through oralcancerprevention.org. The educational cases provided information related to the 5A’s tailored approach to smoking cessation (Ask about smoking, Advise to quit, Assess readiness to quit, Assist quitting (providing individualized skills advice and supportive treatments (nicotine replacement therapy), and Arrange follow-up discussions) and the 5R’s (Relevance, Risks, Rewards, Roadblocks, and Repetition) [9]. Tobacco users in control practices received usual dental care and services. Practices were blinded to the target of the intervention to minimize bias related to the effect of observation on dental practice behavior.

### Pre-intervention dental practice usual care for tobacco Use cessation

Usual care for tobacco use cessation in the dental practices was reported in baseline, pre-intervention, data collection from patients. Each dental was given 100 patient exit cards, and provided instructions on handing out these exit cards to one hundred consecutive adult patients after their visit. These brief postcard-sized surveys were designed to be completed in one to two minutes while the patient was awaiting follow-up instructions and completing payment, and assessed patient tobacco use, age and gender. These exit cards were used to collect baseline data on dental patients and included questions regarding what the dentist said about smoking (ask and advice). Tobacco users who completed the exit cards indicated whether they had been asked about tobacco use, and whether they had been advised to quit if a tobacco user.

### Patient participants and six-month follow-up data

After the http://www.dtc.cme.uab.edu   intervention   was implemented in dental practices each intervention (and comparison usual care control) practice was again given 100 patient exit cards with the same questions on the postcard size survey as at baseline. These cards also asked tobacco users to indicate on the card if they were willing to be contacted for a follow-up call and, if so, provided their name and telephone number. Once all 100 cards were distributed, the dental practice returned the sealed collection box to the study’s coordinating center. Out of the cards returned, 1,361 were from smokers from intervention practices; 1,210 were from smokers from control practices. The dental practices did not have access to the patient responses after being entered into the sealed collection box.

To assess tobacco use cessation, a six-month follow-up telephone survey was conducted among those patients who completed the exit card and indicated willingness to participate by providing a phone number. Participation is summarized in Additional file 2. Patients were called and asked if they would be willing to complete a 10-minute follow-up survey. After verbal consent was obtained, the survey was conducted and each participant was mailed a $10.00 gift card for completing the survey. The six-month follow-up survey confirmed general demographic characteristics, and six-month tobacco use behavior.

Thus, the main dependent (outcome) variable in this report is six-month point prevalent tobacco use abstinence as reported in the follow-up phone survey. The specific question was “Do you smoke cigarettes, cigars, or use smokeless tobacco (dip, chew or snuff) now? (Modification of Behavioral Risk Factor Surveillance System, BRFSS Historical Questions, 2003) We calculated a range of effect sizes for different sample sizes agreeing to participate in follow-up. With the sample size we achieved (over 280 per group), we would have had 80% power to detect a difference of 9% six month tobacco product use quitting, assuming a base rate of quitting of 11%.

### Statistical analyses

The http://www.dtc.cme.uab.edu study was a cluster-randomized trial of a practice-level quality improvement intervention. Thus, the level of randomization was at the practice level and dental patients who used tobacco, the subjects of this analysis, were clustered within these practices. Because cluster-randomized trials are at risk from imbalance of characteristics at levels below the level of randomization (e.g.: patient demographics), we first compared the demographic characteristics of dental patients who used tobacco at baseline recruited from intervention and control practices.

To confirm the impact of our http://www.dtc.cme.uab.edu intervention on dental practices, we then compared patient’s baseline report of their provider performance of asking about tobacco use cessation and advising the tobacco user to quit. We also compared tobacco users’ six-month follow-up reports related to attitudes about tobacco and use of over-the-counter nicotine replacement therapy [11], comparing tobacco users for intervention and control practices.

To test our main hypothesis, that tobacco users seen at http://www.dtc.cme.uab.edu intervention dental practices would have higher rates of six-month tobacco use cessation (as compared with those tobacco users seen at control practices) we began by comparing the proportion of tobacco users reporting point prevalent tobacco use abstinence at six months using a chi-square test. For our primary analysis, we used an intent-to-treat approach [12] including patients regardless of whether they actually reported that their dental provider advised them to quit tobacco. In accordance with current guidelines for analysis of tobacco use cessation trials from the Society for Research on Nicotine and Tobacco Research, our intent-to-treat analysis assigned tobacco users who did not complete six-month follow-up as continued using tobacco (not abstinent) [13]. Because patients were clustered within practices, we accounted for variance inflation due to the clustering by conducting a generalized latent linear and mixed model (GLLMM), with a logit link. The GLLMM regression model was tested with multiple correlation matrices, and was confirmed with a generalized estimating equation. As GLLMM is more robust to variation in cluster size (as in this study), the results from GLLMM are used as the main outcomes. We further adjusted the GLLMM regression model for patient variables (demographic characteristics) that were not balanced in the intervention and control practice patient groups. Again, the main dependent variable was patient-reported six-month point-prevalent tobacco use cessation abstinence and the independent variable was intervention or control practice status. All analyses were conducted using the STATA statistical program, version 11.

## Results

### Practice characteristics and usual care (screening for smoking and advising smokers to quit)

Most of the 143 practices were general dentistry practices (92%) and solo practices (79%). Practices were located in Alabama (25%), Florida (34%), Georgia (27%), and North Carolina (14%). Overall, these 143 practices included 185 dentists (89 intervention and 96 control) and 274 hygienist participants (137 intervention and 137 control). Pre-intervention data collected from patient exit cards indicates that usual care in these practices varied considerably. As reported on the exit cards pre-intervention, these 143 practices screened for smoking status (at that visit) a mean of 28% of patients (standard deviation 19% (range 3%-91%). Practices advised 41% of known smokers to quit (standard deviation 21%). Comparing usual care in intervention and control practices, we found no significant difference in mean screening rates (29% intervention, 28% control, p = 0.5) or advice to quit (42% intervention, 40% control, p = 0.6). Thus, although the usual care varied, it was balanced across the intervention and control groups.

### Patient characteristics

From the 143 participating practices, 564 patients completed six-month follow-up, (See Table 1). There were no significant differences between the control and intervention patients for sex, race, and education or for living with a smoker. Significant differences (p = .03) emerged for ethnicity among the patients, with 2% in the Intervention group self-reporting to be of Hispanic origin and 5% in the Control group. Significant differences also emerged for general health items (p = .04), with patients in the Intervention Group self-reporting lower levels of excellent, very good and good health compared to patients in the Control group (See Table 1).

**Table 1:** Dental practice patient population characteristics*

	**Intervention**	**Control**	**p +**
	n/N (%)	n/N (%)	
**Age**			**0.125**
**19-29**	66/315 (21%)	54/261 (21%)	
**30-44**	129/315 (41%)	78/261 (30%)	
**45-59**	86/315 (27%)	100/261 (38%)	
**60+**	34/315 (11%)	29/261 (11%)	
**Sex**			**0.164**
**Male**	150/312 (48%)	110/261 (42%)%)	
**Female**	162/312 (52%)	151/261 (58%)	
**Ethnicity**			**0.028**
**Hispanic/Latino**	4/250 (2%)	11/209 (5%)	
**Race**			**0.213**
**White**	216/251 (86%)	174/209 (83%)	
**African America**	32/251 (13%)	19/209 (9%)	
**Asian**	1/251 (0%)	5/209 (2%)	
**General Health**			**0.042**
**Excellent**	38/250 (15%)	38/210 (18%)	
**Very Good**	86/250 (34%)	81/210 (39%)	
**Good**	81/250 (32%)	72/210 (34%)	
**Fair/Poor**	45/250 (18%)	19/210 (9%)	
**Education**			**0.616**
**Elementary school (1–8)**	3/252 (1%)	2/211 (1%)	
**Some High School (9–11)**	26/252 (10%)	15/211 (7%)	
**High School Graduate (12 or GED)**	76/252 (30%)	61/211 (29%)	
**Some College (1–3 years)**	84/252 (33%)	69/211 (33%)	
**College Graduate (4 years or more)**	63/252 (25%)	64/211 (30%)	
**Lives with smoker**	90/251 (36%)	83/210 (40%)	**0.418**

*Total denominator varies due to some missing data for some patient characteristics, + Pearson chi2 or Mantel Haenszel chi2 for trend used as appropriate.

### Patient reports of dental provider performance of asking and advising tobacco users to quit

Confirming the impact of our http://www.dtc.cme.uab.edu intervention [9], tobacco users in the intervention group more frequently reported that their dental provider had asked and advised tobacco users to quit (see Table 2). These reports occurred on the exit cards completed immediately after their dental visit for those patients seen after the intervention.

**Table 2:** Patient reports of advice during dental visits, comparing post-intervention Oralcancerprevention.org and comparison control*

**Item**	**Intervention**	**Control**	**P**
Gave advice to quit tobacco use	181/330 (55%)	106/273 (39%)	**0.001**
Gave Written advice	83/253 (33%)	47/211 (22%)	**0.012**

*Total denominator varies due to some missing data for some patient characteristics.

**Table 3:** Patient perception on health risk of tobacco products, use of NRT and six-month quitting of tobacco product use

**Item**	**Intervention**	**Control**	**P**
Views Smoking as Health Risk			**0.410**
Strongly Agree	202/251 (81%)	163/211 (77%)	
Agree	42/251 (17%)	44/211 (21%)	
Neutral	5/251 (2%)	4/211 (2%)	
Disagree/Strongly Disagree	2/251 (1%)	0/211 (0%)	
Oral Health Risk			**0.370**
Strongly Agree	163/251 (65%)	133/211 (63%)	
Agree	77/251 (31%)	65/211 (31%)	
Neutral	7/251 (3%)	12/211 (6%)	
Disagree/Strongly Disagree	2/251 (1%)	1/211 (0%)	
Don’t know/not sure	2/251 (1%)	0/251 (0%)	
Nicotine Replacement Therapy Use	4/26 (15%)	6/31 (19%)	**0.695**
Quit tobacco products at six months(1)	30/340 (9%)	37/283 (13%)	**0.088**

### Attitudes about tobacco and use of nicotine replacement at follow-up

At six month of follow-up, we found no differences in patient-reported attitudes of smoking, comparing patients seen at intervention and control practices. We also noted no difference in using nicotine-replacement therapy in the two groups (Table 2).

### Main outcome - Six-month point prevalent tobacco use cessation

In unadjusted analysis of six-month point prevalent quitting tobacco products, 9% of patients seen by http://www.dtc.cme.uab.edu intervention practice providers, compared with 13% among those seen in control practices. This 4% difference in cessation, actually favoring patients seen in the usual care control interventions nearly approached statistical significance (p = 0.088) (See Table 3).

In bivariate logistic regression, control practice patients were again more likely to quit tobacco products compared to intervention (Unadjusted Odds Ratio 0.643 (95%    CI   0.39  – 1.07)   p = 0.09).    After    adjusting    for clustering of tobacco users within dental practices, and adjusting for patient self-reported health, a variable significantly different in the intervention and control, we found no difference in the six-month follow-up report of tobacco use cessation, comparing intervention and control group. Tobacco users seen in intervention practices were no longer significantly less likely to report tobacco use cessation (Odds Ratio = 0.76 (0.44-1.30) p = 0.323).

### Stratified analyses and quit attempts

In stratified analyses, among those with fair-poor health, tobacco users from intervention practices were not significantly more likely to quit as compared with control (Odds Ratio 1.75 (95% CI 0.2-16) p = 0.6), although the direction of the point estimate was positive. Among those in good or excellent health, intervention tobacco users were again not significantly likely to quit (Odds Ratio 0.7 (95% CI 0.4-1.26), p = 0.25), but the point estimate followed that of the main analysis.

Among those that did not quit using tobacco, 46% reported at least one quit attempt (ranging from 1 to 4 attempts), and the proportion with at least one quit attempt was not different among intervention and control patients (46.5% versus 45%, p = 0.6).

## Discussion

In this report of patients from 143 community-based dental practices across multiple states, we found that a successful real-world, web-assisted tobacco control quality improvement program resulted in improvement in dental practice provision of brief tobacco use cessation advice. However, this practice improvement intervention was not strong enough to result in differentially higher rates of six-month tobacco use cessation among tobacco users seen at practices participating in the http://www.dtc.cme.uab.edu intervention, compared with usual-care control practices. Our evaluation adds considerably to the literature, extending the possibility to change provider behavior and the challenges of changing patient behavior.

Prior Dental tobacco interventions have had varied affects. A Cochrane review published in 2012 reported of 14 interventions in dental settings, pooled results suggested these interventions can increase tobacco abstinence rates (odds ratio [OR] 1.71, 95% confidence interval [CI] 1.44 to 2.03) at six months or longer, but with notable heterogeneity of effect [14]. Gordon and colleagues provided in-person 5As training in one public health, and compared with one non-randomized control, and found that Patients in the intervention group were more likely to quit than those receiving usual care (15.5 versus 4.3 percent) and after 12 months (18.8 versus 4.6 percent) [15]. Attempting to integrate a tobacco Quitline into dental clinics increased tobacco cessation in some, but few dental patients were engaged with the Quitline [16]. The majority of interventions reported in the above Cochrane collaboration review provided more intensive training for dental practices, and thus include a small number of practices (with an mean patients per study of 750) [http://14]. In contrast, our study recruited 143 dental practices and used a pragmatic, technology-assisted approach to training (light touch, high tech). Thus, our study had a greater reach, achieved a significant difference in provider behavior, but was not able to demonstrate an impact on dental patient behavior.

Despite the evidence of tobacco risk and the efficacy of tobacco control interventions, 30% of current smokers report that they have never been advised to stop smoking by a healthcare provider [17]. As discussed, the dental practice provides an opportune time to speak with tobacco users about the dangers associated with tobacco (both smoking and smokeless tobacco), and also provide advice on ways to quit. In their care, dentists and hygienist already provide advice regarding flossing, teeth brushing and oral hygiene, so it is a natural setting for patients to receive advice about another health issue that influences oral hygiene and care. Increasing delivery of tobacco use cessation counseling and related tobacco control practices at point of care is necessary to increasing the impact of dental providers on the behavior of their patients. Although we successfully increased provision of tobacco use cessation advice in intervention dental practices, this improvement was not sufficient to actually change the behavior of the patients exposed to the improvement practices.

The goal of a randomized trial is to demonstrate the causal relationship between an intervention exposure and an outcome of interest. The benefit of randomization is to create balance in measured and unmeasured characteristics in the intervention and control groups, thereby isolating the intervention effect. Cluster-randomized trials are imperfect in their ability to produce balance of measured and unmeasured characteristics at levels below the level of randomization. Despite the large number of dental practices randomized in this study, we found a clear imbalance in patient characteristics between the two groups. Without adjustment, we might have concluded that increasing rates of tobacco use cessation advice at the point of care in dental practices resulted in *lower* cessation rates, approaching statistical significance. However, this was clearly confounded by the higher self-reported illness in the patients seen in http://www.dtc.cme.uab.edu intervention practices and who agreed to participate in follow-up. This may suggest that our practice-level quality improvement intervention resulted in providers reaching out to sicker tobacco users who may be more difficult to persuade to quit. After adjustment, we appropriately found no difference at all in the intervention or control. Future cluster-randomized trials need to again carefully measure patient characteristics that may be imbalanced and confound results in the trials.

### Limitations

Our http://www.dtc.cme.uab.edu results are important, despite the patient-level p-value, as they highlight the difficulty associated with changing tobacco behavior. While many interventions do exist, we still need innovations. Thus, the results from this study provide useful and cautionary results. Interventions targeted at the dental practice level, may be more effective if paired with patient-level interventions designed to provide continued support after the dental visit. We had a limited number of tobacco users per practice available for follow-up, limiting the precision of our estimates. As noted, our cluster-randomized trial did not achieve balance in patient-level characteristics, lending to somewhat limited generalizability. In addition to our main conclusion that quality improvement interventions like http://www.dtc.cme.uab.edu may be necessary but not sufficient to impact dental patient behavior; our analysis provides an interesting specific example of the potential problems of cluster-randomization.

## Conclusion

Tobacco control quality improvement programs can increase performance of brief cessation advice in dental practice, but patient-targeted interventions may be necessary to achieve meaningfully higher rates of cessation. Because the dental visit is an opportune time to discuss oral health and how smoking affects their oral and overall health, interventions should continue to target dental practices. Continued work in methods for delivery of individualized tobacco use cessation counseling meaningfully related to oral health, and how this information is presented to patients is needed. Studies that integrate provider and patient-targeted intervention are also needed.

## Competing interest

The authors declare that they have not competing interests.

## Authors’ contributions

TH worked directly in the planning, and design of the implementation to include the intervention website, was directly involved in all analyses of data. KD was involved in the analysis and creation of the manuscript. MR was involved in the design and implementation of the study. GG was involved in the creation of the study and participated in its design and coordination of implementation. JA was involved in the analyses and interpretation of study findings. CK was involved in the design of the study. JV was involved in manuscript creation and study analyses. All authors edited and approved the final manuscript.

## Authors’ information

The National Dental PBRN Collaborative Group comprises practitioners, faculty and staff who contributed to this DPBRN activity. A list of these persons is at http://nationaldentalpbrn.org/publication.php.

## Pre-publication history

The pre-publication history for this paper can be accessed here:

http://www.biomedcentral.com/1472-6831/13/13/prepub

## Supplementary Material

Additional file 1 Appendix A. Click here for file

Additional file 2 **Appendix B.** Consort Diagram DTC Patient Participation. Click here for file

Additional file 3 CONSORT 2010 checklist of information to include when reporting a randomised trial*. Click here for file
