# Supplementary material for: Cluster-randomized trial of a web-assisted tobacco quality improvement intervention of subsequent patient tobacco product use: a National Dental PBRN study
Source: BMC Oral Health. 2013 Feb 23;13:13. doi: 10.1186/1472-6831-13-13 (PMC3623865; doi:10.1186/1472-6831-13-13)
Supplement: Additional file 1 — Appendix A. [file 1472-6831-13-13-S1.pdf]

Appendix A

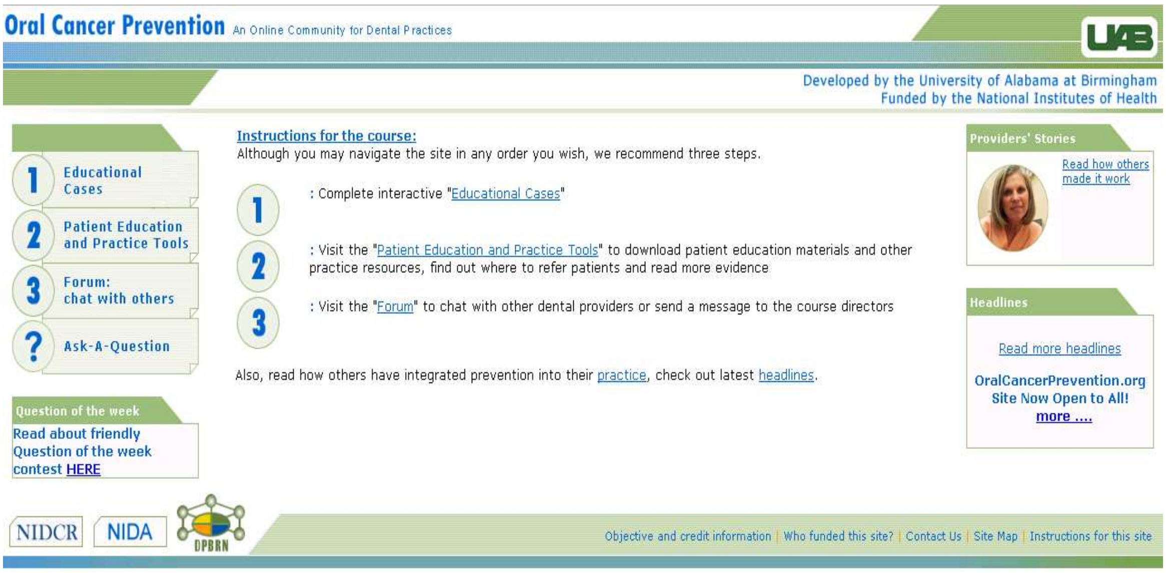
Oral Cancer Prevention An Online Community for Dental Practices
UAB
Developed by the University of Alabama at Birmingham
Funded by the National Institutes of Health
1 Educational Cases
2 Patient Education and Practice Tools
3 Forum: chat with others
? Ask-A-Question
Question of the week
Read about friendly Question of the week contest HERE
Instructions for the course:
Although you may navigate the site in any order you wish, we recommend three steps.
1 : Complete interactive "Educational Cases"
2 : Visit the "Patient Education and Practice Tools" to download patient education materials and other practice resources, find out where to refer patients and read more evidence
3 : Visit the "Forum" to chat with other dental providers or send a message to the course directors
Also, read how others have integrated prevention into their practice, check out latest headlines.
Providers' Stories
Read how others made it work
Headlines
Read more headlines
OralCancerPrevention.org Site Now Open to All!
more ....
NIDCR
NIDA
DPBRN
Objective and credit information | Who funded this site? | Contact Us | Site Map | Instructions for this site
