# Supplementary figures and images for: Cluster-randomized trial of a web-assisted tobacco quality improvement intervention of subsequent patient tobacco product use: a National Dental PBRN study
Source: BMC Oral Health. 2013 Feb 23;13:13. doi: 10.1186/1472-6831-13-13 (PMC3623865; doi:10.1186/1472-6831-13-13)

Appendix B: Consort Diagram DTC Patient Participation

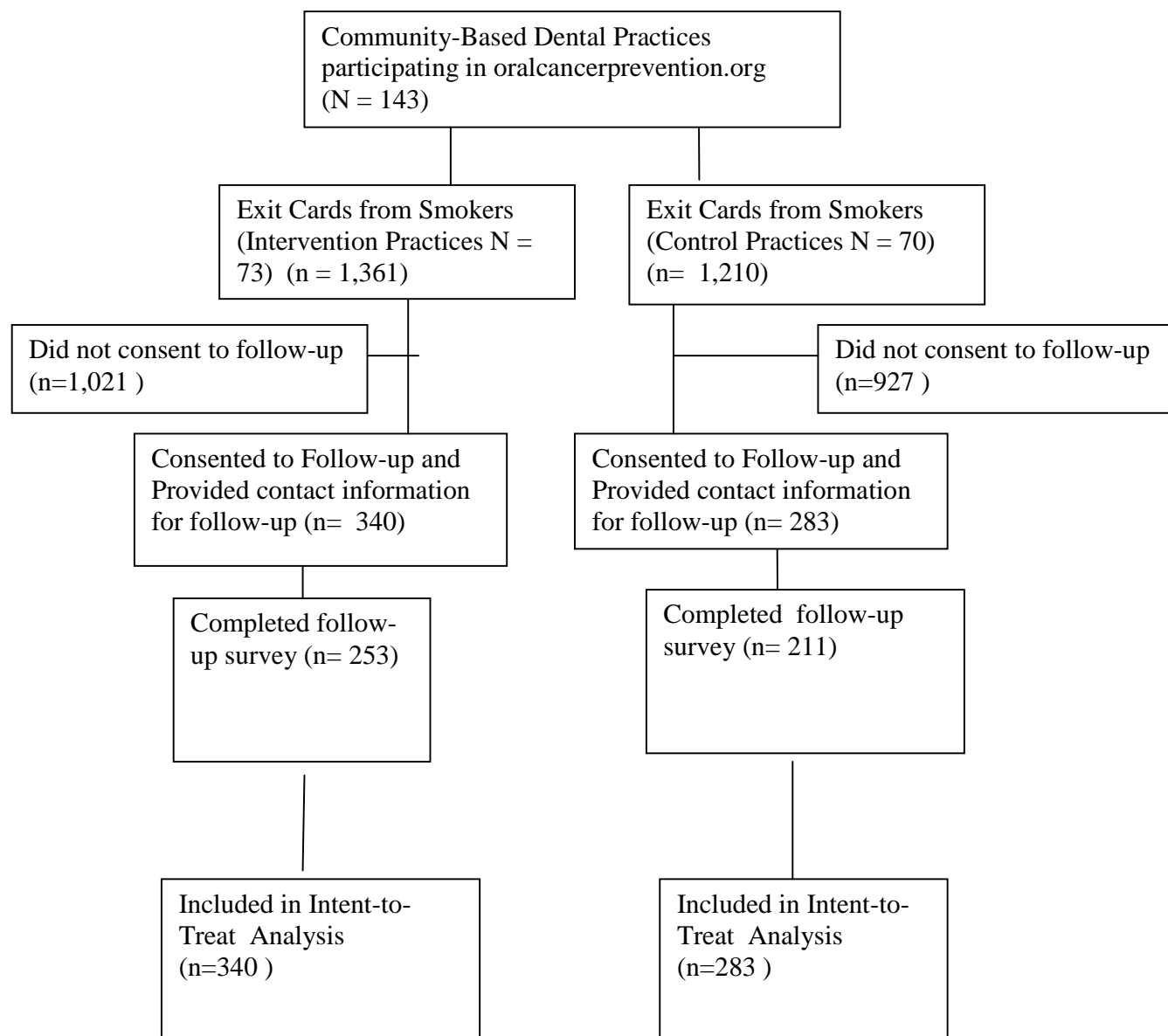

Supplement: Additional file 2 — Appendix B. Consort Diagram DTC Patient Participation. [file 1472-6831-13-13-S2.pdf]
